# Dentistry in Brazil

**Published:** 1895-11

**Authors:** Thos. B. Mercer

**Affiliations:** Minneapolis


### Dentistry in Brazil.

BY THOS. B. MERCER, D.D.S., MINNEAPOLIS.

Mr. Ward McAllister, in his treatment of the mechanism of society, very wisely named his work "Society as I Have Found It," so that, I take it, criticism could be attributed to personal opinion rather than as a statement of absolute fact. I will plagiarize the idea, and consider my article "Dentistry in Brazil as I Have Found It" in all but title, for the same reason.

To review dentistry in the Brazilian Republic, it may be well, in view of the fact that it may be considered on open field for location to speak of its advantages and disadvantages as such in addition to a general description.

The climate, an all-important factor to the foreigner, is of course, distinctly tropical, accompanied by that lavish display of vegetation characteristic of the tropics—becoming in the lowlands a perfect net-work of palms, mosses, grasses, etc., luxuriant beyond description. The summer season, from about December 1st to April, is extremely hot and as the large cities are all seaport towns (with the exception of Sao Paulo), low and damp, they become veritable incubators for low-fever germs which, to the unacclimated new-comer, is often fatal, but in other seasons it is often pleasant indeed and until the novelty wears off is really quite ideal.

The people, a mixtnre of the Portuguese, African negro and native Indian, are an olive complexioned, rather slight people—the better class pleasant to meet, quite cordial, but inclined to be hypocritical. Their government is at present a very unstable affair, which is detrimental to money exchange and cripples the country. For admittance to practice dentistry in your own name an examination in the Portuguese language before the Medical Board of Rio de Janeiro must be taken which, by the way, is very severe, but by being in the employ of or using the name of some one licensed, you are not interfered with.

Practically all of the representative dentists are North Americans who employ from three to five more, but there are some native practitioners who come to "the States" for their course, then return. Prices being high the work among these representatives is of a very superior quality and conscientiously done. Their offices are fully equipped with all late appliances, but there are native dentists who are about as numerous as barbers and, who, with few exceptions, maintain about the same degree of dignity. Their outfit would include little more than forceps, amalgam, a spatula and arsenic. They use the latter about as freely as we would dental plaster—from as an obtundent in a simple cavity to an exposure. The pulp is never removed—so when pulpitis or an abscess develops it is extracted or they "save up their money" and apply to a "dentista Americana" whose appointment book usually has a dozen or more cases bearing evidence of the skill(?) of these native operators. Outside of the office the life is hardly as agreeable as most of us would wish, as for the first year you are handicapped by the languague and after you have mastered that after a fashion, and feel that you would like to become acquainted and mingle somewhat in the society that is afforded, you are confronted with the fact that, humiliating as it may seem, a dentist holds an inferior position. This also holds good among the British residents. But grant that you gain the dental entrie, you soon find their ways and what is expected of you so different from what you are accustomed to that you are pleased to let it alone and return to your dental friends once more, where, with the exception of a dinner or an excursion "up country" now and then you experience the steady routine of work, eat and sleep.

This, however, may be a meager argument to many, when I repeat that prices are good, and there is usually plenty of work, after you are once established, but after some little experience the question arises to me: "Is the game worth the candle?"

